# Rapid inactivation and sample preparation for SARS-CoV-2 PCR-based diagnostics using TNA-Cifer Reagent E

**DOI:** 10.3389/fmicb.2023.1238542

**Published:** 2023-10-06

**Authors:** Nina M. Pollak, Daniel J. Rawle, Kexin Yan, Cameron Buckley, Thuy T. Le, Claire Y. T. Wang, Nicole G. Ertl, Karla van Huyssteen, Nicole Crkvencic, Misha Hashmi, Russell E. Lyons, David M. Whiley, Andreas Suhrbier, Joanne Macdonald

**Affiliations:** ^1^Center for Bioinnovation, University of the Sunshine Coast, Sippy Downs, QLD, Australia; ^2^School of Science, Technology and Engineering, University of the Sunshine Coast, Sippy Downs, QLD, Australia; ^3^DMTC Limited, Kew, VIC, Australia; ^4^Inflammation Biology Group, QIMR Berghofer Medical Research Institute, Herston, QLD, Australia; ^5^Faculty of Medicine, UQ Centre for Clinical Research, The University of Queensland, Herston, QLD, Australia; ^6^Queensland Paediatric Infectious Diseases Laboratory, Centre for Children's Health Research, Brisbane, QLD, Australia; ^7^BioCifer Pty Ltd., Auchenflower, QLD, Australia; ^8^Bio Molecular Systems, Potts Point, NSW, Australia; ^9^Microbiology Department, Pathology Queensland, Herston, QLD, Australia; ^10^GVN Center of Excellence, Australian Infectious Disease Research Centre, Herston, QLD, Australia

**Keywords:** COVID-19, SARS-CoV-2, RT-qPCR, diagnostics, virus inactivation, safety, RNA extraction

## Abstract

RT-qPCR remains a key diagnostic methodology for COVID-19/SARS-CoV-2. Typically, nasal or saliva swabs from patients are placed in virus transport media (VTM), RNA is extracted at the pathology laboratory, and viral RNA is measured using RT-qPCR. In this study, we describe the use of TNA-Cifer Reagent E in a pre-clinical evaluation study to inactivate SARS-CoV-2 as well as prepare samples for RT-qPCR. Adding 1 part TNA-Cifer Reagent E to 5 parts medium containing SARS-CoV-2 for 10 min at room temperature inactivated the virus and permitted RT-qPCR detection. TNA-Cifer Reagent E was compared with established column-based RNA extraction and purification methodology using a panel of human clinical nasal swab samples (*n* = 61), with TNA-Cifer Reagent E showing high specificity (100%) and sensitivity (97.37%). Mixtures of SARS-CoV-2 virus and TNA-Cifer Reagent E could be stored for 3 days at room temperature or for 2 weeks at 4°C without the loss of RT-qPCR detection sensitivity. The detection sensitivity was preserved when TNA-Cifer Reagent E was used in conjunction with a range of VTM for saliva samples but only PBS (Gibco) and Amies Orange for nasal samples. Thus, TNA-Cifer Reagent E improves safety by rapidly inactivating the virus during sample processing, potentially providing a safe means for molecular SARS-CoV-2 testing outside traditional laboratory settings. The reagent also eliminates the need for column-based and/or automated viral RNA extraction/purification processes, thereby providing cost savings for equipment and reagents, as well as reducing processing and handling times.

## 1. Introduction

The emergence of the novel severe acute respiratory syndrome coronavirus 2 (SARS-CoV-2) in December 2019 led to the rapid global spread of coronavirus disease 2019 (COVID-19). As of June 2023, there have been over 760 million cases, with more than 6.8 million deaths worldwide. This has led the increased awareness that COVID-19 can lead to long-lasting effects, which can be referred as long-COVID (Davis et al., 2023). Rapid and reliable diagnostics represent a key intervention worldwide and also have informed *inter alia* public health measures, treatment options, and vaccination strategies. Although rapid antigen tests are currently available, they often have poor sensitivity compared to reverse transcription-quantitative polymerase chain reaction (RT-qPCR) (Li et al., 2023), especially for new variants of concern (Mohammadie et al., 2023) and during the early onset of infection (Jeewandara et al., 2022; Puhach et al., 2023). Failure to promptly diagnose COVID-19 during symptomatic infection would delay the initiation of treatment, such as nirmatrelvir/ritonavir (Paxlovid), thereby compromising its effectiveness for vulnerable individuals (Wang et al., 2023).

RT-qPCR has remained the cornerstone for diagnosing COVID-19, with a vast array of innovative approaches emerging during the outbreak. Notably, the use of pooled testing strategies along with the implementation of suitable algorithms have increased the testing power without increasing resource requirements (Ivan et al., 2021; Song et al., 2022). Improvements in ergonomics are exemplified by the Istanbul airport, with a per day capacity of 20,000 tests and a rapid 1-h turnaround time (Istanbul-Airport). Newer systems, such as GeneXpert, have been developed, requiring less extensive training, infrastructure, and equipment, making them well-suited for resource-limited settings (Oladimeji et al., 2020; Rakotosamimanana et al., 2020). There has been a surge in innovative sample preparation methods, such as, strip- or cartridge-based systems for the aforementioned PCR machines and new kits such as the QIAprep&amp Viral RNA UM kit (Becerril Vargas et al., 2022). Increasing safety has been a key objective, with various developments aimed at virus inactivation before testing, thereby mitigating the risk for laboratory staff and reducing the need for equipment, infrastructure, and practices (e.g., containment level 2 requirements) that maintain infection control for a virus whose predominant transmission mode is via aerosol (Geng and Wang, 2023). For example, heating has been widely reported for inactivation and RNA extraction (Ranoa et al., 2020; Vogels et al., 2021; Dewhurst et al., 2022), although this requires heating equipment and appropriate temperature monitoring and can result in decreased sensitivity (Delpuech et al., 2022).

A simple new sample preparation reagent, TNA-Cifer Reagent E, has been described for use in RT-qPCR-based detection in a number of infectious disease settings (Ahmed et al., 2022a,b; Pollak et al., 2022a,b, 2023a,b). In this study, we describe a pre-clinical evaluation of the use of this reagent for the RT-qPCR-based detection of SARS-CoV-2. When mixed with SARS-CoV-2-containing samples, TNA-Cifer Reagent E rapidly inactivated the virus and extracted the viral RNA. Thus, TNA-Cifer Reagent E/sample mixtures can be added directly to RT-qPCR reactions without the need for any other RNA extraction or purification processes.

## 2. Materials and methods

### 2.1. Ethics statement and approvals

The study was approved by the Human Research Ethics Committee of the Royal Children's Hospital, including for the use of deidentified human nasal swabs from suspected COVID-19 patients (Enhanced Characterization of Respiratory Virus Infections LNR/19/QCHQ/49476).

All research involving infectious SARS-CoV-2 was conducted in the BioSafety Level 3 (PC3) facility at the QIMR Berghofer MRI (Australian Department of Agriculture, Fisheries and Forestry certification Q2326 and Office of the Gene Technology Regulator certification 3445).

### 2.2. SARS-CoV-2, propagation, UV-inactivation, and quantitation

An original (ancestral) strain patient isolate, SARS-CoV-2_QLD02_ (hCoV-19/Australia/QLD02/2020), was generously provided by Drs. A. Pyke and F. Moore (Queensland Health Forensic and Scientific Services, Queensland Department of Health, Brisbane, Australia). We collected whole genome sequences deposited at GISAID, Accession ID: EPI_ISL_407896 (https://gisaid.org/) and GenBank, Accession ID: MW772455.1. The virus was propagated in Vero E6 cells as described (Yan et al., 2021, 2022), with the culture medium tested for endotoxin (Johnson et al., 2005), and then, the Vero E6 cells and viral stocks were tested for mycoplasma (MycoAlert Mycoplasma Detection Kit MycoAlert, Lonza) (La Linn et al., 1995). Viral titers were determined by CCID_50_ assays (Yan et al., 2021, 2022). UV inactivation and confirmation were undertaken by CCID_50_ assays as described in studies by Yan et al. (2021), Morgan et al. (2022), and Yan et al. (2022). The number of RNA copies/μl was calculated by titrating using the “Twist Synthetic SARS-CoV-2 RNA Control 2 (MN908947.3)” (Decode Science, Mount Waverly, Victoria) and RNA from UV-inactivated SARS-CoV-2 isolated using the NucleoSpin RNA, Mini Kit for RNA purification (Macherey-Nagel, Dueren, Germany). The virus stock at 7.5 log_10_CCID_50_/ml contained 15,475,000 RNA copies/μl. Two viral stocks were prepared for these studies, one at 7.3 log_10_CCID_50_/ml and the other at 7.5 log_10_CCID_50_/ml.

### 2.3. TNA-Cifer Reagent E

The TNA-Cifer Reagent E was supplied by BioCifer Pty. Ltd. (Auchenflower, Qld., Australia), which is ISO 9001 certified. TNA-Cifer Reagent E is a liquid reagent designed to be mixed with samples to enable pathogen inactivation, nucleic acid extraction, and PCR inhibitors' removal in a single-step reaction. The reagent has the following GHS classifications: Flammable liquid (Category 2), H225; skin corrosion/irritation (Category 1), H314; specific-target organ toxicity—single exposure (Category 3), H336.

### 2.4. Inactivation of SARS-CoV-2 with TNA-Cifer Reagent E

The TNA-Cifer Reagent E was mixed with stocks of SARS-CoV-2 in a medium (RPMI 1640 supplemented with 2% FCS; Sigma-Aldrich, St Louis, USA) at the indicated ratios (1 in 6 and 1 in 10) and incubated at room temperature for a specific duration. After being diluted in the medium (RPMI 1640, 2% FCS), mixtures were titrated in duplicate using 10-fold serial dilutions in 96-well plates. Vero E6 cells (ATCC, CRL-1586) in 100 μl of the medium (RPMI 1640 with 10% FCS) were then added (10^4^/well). After 4 days, plates were formalin-fixed and stained with crystal violet and OD_595nm_, measured as described by Yan et al. (2021). High OD represents no virus-induced cytopathic effects; low OD indicates viral replication-induced cytopathic effects.

### 2.5. RT-qPCR; TNA-Cifer Reagent E vs. NucleoSpin RNA virus kit

SARS-CoV-2 RNA was extracted using TNA-Cifer Reagent E or the NucleoSpin RNA virus kit (Macherey-Nagel, Dueren, Germany), as per the manufacturer's instructions, except for input and output volumes, which were 20 μl; no carrier RNA was used (Figure 2). RT-qPCR was undertaken as described by Rawle et al. (2021) and Dumenil et al. (2023). Briefly, samples containing viral RNA (5 μl) were added to 15 μl iTaq Universal Probes One-Step Kit (BioRad; Hercules, CA, USA), and RT-qPCR was undertaken as per the manufacturer's instructions. The E-Sarbeco primer set (400 nM for each; Integrated DNA Technologies Australia, Sydney, Australia) (Vogels et al., 2020) and the E-Sarbeco P1 5′ FAM—ZEN™/Iowa Black^®^ FQ probe (20 nM;Integrated DNA Technologies Australia, Sydney, Australia) were used for the PCR. Reactions were placed into a BioRad CFX96 based on the following cycling protocol: 10 min at 50°C, 3 min at 95°C, and 40 cycles of 14 s at 95°C and 30 s at 60°C. PCR products were confirmed by gel electrophoresis (1% agarose) with an expected fragment size of 125 bp.

### 2.6. RT-qPCR of patient nasal swab samples; MagNA pure vs. TNA-Cifer Reagent E

Deidentified frozen human nasal swab samples in the Sigma-Virocult^®^ virus/specimen transport medium (Sigma) were provided by Pathology Queensland (n=41 containing known positives, and n=20 all determined to be negative). The samples were thawed and RNA-extracted using either (i) the MagNA Pure96 DNA and Viral NA Small Volume kit with the MagNA Pure 96 Instrument (Roche Diagnostics, Basel, Switzerland) using the Pathogen Universal 200 Protocol or (ii) TNA-Cifer Reagent E. RNA samples (5 μl) were added to an RT-qPCR master mix (15 μl) containing the SensiFAST™Probe Lo-ROX One-Step kit (Meridian Bioscience, Cincinnati, USA), China CDC ORF1ab primers (F: CCCTGTGGGTTTTACACTTAA, R: ACGATTGTGCATCAGCTGA), and the 6FAM- CCGTCTGCGGTATGTGGAAAGGTTATGG-BHQ1 probe (Niu et al., 2020). RT-qPCR was run using a Rotor-Gene 6000 or a Rotor-Gene Q thermal cycler (Qiagen, Hilden, Germany).

### 2.7. Storage of SARS-CoV-2 in TNA-Cifer Reagent E for different times and temperatures

UV inactivation of SARS-CoV-2 permitted the release of SARS-CoV-2 from PC3/BSL3 containment, allowing this experiment to be performed under PC2/BSL2 biocontainment conditions. The virus stock before inactivation was 7.5 log_10_CCID_50_/ml. The UV-inactivated virus was serially diluted in RPMI 1640 supplemented with 2% FCS. TNA-Cifer Reagent E was added (20 μl sample plus 4 μl reagent) and incubated for 10 min, 90 min, 5 h, 24 h, 72 h, 1 week, or 2 weeks at room temperature, 4°C or −20°C (single-freeze thaw). At the indicated times, 5 μl of the mixture was added in duplicate to the RT-qPCR master mix (15 μl). RT-qPCR was undertaken using E-Sarbeco primers, as described above.

### 2.8. Evaluation of VTM compatibility with TNA-Cifer Reagent E

The following VTM and swab systems were evaluated: Water (UltraPure Distilled Water, Invitrogen, Cat#10977-023), RPMI (RPMI 1640 supplemented with 2% fetal bovine serum and antibiotics, Sigma-Aldrich), Virocult (Sigma-Virocult, Medical Wire and Equipment, Cat# MW951S; Wiltshire, UK), Amies Blue (Sigma Transwab 1ml liquid Amies/Light Blue Cap/1x Pureflock Ultra Fine Swab, Medical Wire and Equipment, Cat# MW178PF), Amies Orange (Sigma transwab 2ml liquid Amies/Orange Cap, Standard Sigma Swab, Medical Wire and Equipment, Cat# MW176S0), UTM (Universal Transport Medium, Copan Diagnostics, Cat# 330C-3ML; Murrieta, CA, USA), saline (Baxter Sodium Chloride 0.9 % for irrigation sterile saline-−100ml—Bottle–Each, Alpha Medical Solutions, Cat# AHF7975; St Ives, NSW, AU), PBS (Gibco; 5 g Gibco^®^ PBS tablet dissolved in 500 ml of distilled water, pH 7.45, Gibco, Cat# 18912014; Thermo Fisher Scientific Australia, Scoresby VIC), and PBS (Edwards; Phosphate buffer solution−99 ml, MicroMedia, Edwards Group, Cat# OPM 90; Narellan NSW, AU). Virocult, Amies, and UTM came with their own swabs; for the remaining VTM, Minitip FLOQ swabs available in sachets were used (Copan Diagnostics, Cat# 501CS01). The UV-inactivated virus was diluted in VTM, VTM plus nasal swab samples from healthy volunteers, or VTM plus saliva swab samples from healthy volunteers. The dilutions were then added to TNA-Cifer Reagent E and analyzed by RT-qPCR alongside the virus diluted in RPMI 1640 plus 2% FCS (RPMI control) using E-Sarbeco primers.

### 2.9. Use of TNA-Cifer Reagent E with Bio Molecular Systems' reagents and PCR cycler

Sixteen frozen and deidentified combined naso/oropharyngeal swab samples suspended in PBS were tested, they were collected from patients who tested positive for SARS-CoV-2 on PCR testing, and the samples were obtained from a large pathology provider in NSW. Traditional RNA purification was performed with the MagMAX™ Total Nucleic Acid Isolation kit (Thermo Fisher Scientific Australia, Scoresby VIC), using a 200-μl sample combined with Proteinase K, followed by magnetic bead isolation with a final eluate volume of 50 μl. The RT-qPCR setup was performed using the Myra automatic robotic handler [Bio Molecular Systems, BMS, Upper Coomera, QLD, AU (Myra-Website)], which mixed 10 μl eluate, 4.5 μl proprietary BMS master mix (containing reverse transcriptase, Taq DNA polymerase, and co-factors in buffer), and 5.5 μl of a proprietary BMS SARS-CoV-2 oligonucleotide mix (containing primers and hydrolysis probes targeting the RNA-dependent RNA-polymerase and nucleocapsid gene regions of SARS-CoV-2, along with a human RNase P internal control). Fast RT-qPCR was performed on the Mic cycler (BMS), according to the following optimized cycling protocol: reverse transcription for 3 min at 50°C; initial activation for 30 s at 95°C; 5 pre-cycles of denaturation for 1 s at 95°C and annealing for 5 s at 65°C; and 40 cycles of denaturation for 1 s at 90°C and annealing for 1 s at 65°C, with time duration of 31 min and 2 s for completion of the process.

Direct to PCR QIAprep&amp, the Viral RNA UM kit (Qiagen Hilden, Germany) was also performed using the BMS Myra, which mixed 2 μl of an inactivating UM prep buffer with 8 μl sample, paused to incubate samples for 2 min at room temperature, and then added 5 μl Qiagen master mix and 5.5 μl proprietary BMS SARS-CoV-2 oligonucleotide mix. Fast RT-qPCR was performed on the Mic cycler (BMS) according to the following optimized cycling protocol: reverse transcription for 10 min at 50°C; initial activation for 2 min at 95°C; 5 pre-cycles of denaturation for 5 s at 95°C and annealing for 20 s at 65°C; and 40 cycles of denaturation for 5 s at 90°C and annealing for 20 s at 65°C, with time duration of 49 min and 47 s for completion of the process.

TNA-Cifer Reagent E sample preparation and RT-qPCR preparation were also performed on the BMS Myra, which mixed 5 μl sample with 1 μl TNA-Cifer Reagent E (BioCifer Auchenflower QLD), paused to incubate samples for 5 min at room temperature, and then added 5 μl Ampli-Cifer RT-qPCR mix (BioCifer); 5.5 μl proprietary BMS SARS-CoV-2 oligonucleotide mix; and 3.5 μl nuclease-free water. Fast RT-qPCR was performed on the Mic cycler (BMS) according to the following optimized cycling protocol: Reverse transcription for 5 min at 50°C; initial activation for 2 min at 95°C; 5 pre-cycles of denaturation for 5 s at 95°C and annealing for 20 s at 65°C; and 40 cycles of denaturation for 3 s at 90°C and annealing for 15 s at 65°C, with time duration of 46 min and 47 s for completion of the process.

All RT-qPCR results from the BMS Mic qPCR device were analyzed using the accompanying micPCR software (BMS), which reports internal control normalized Cq values and efficiency measures.

### 2.10. Statistics

Statistical analyses of the experimental data were performed using IBM SPSS Statistics for Windows, Version 19.0 (IBM Corp., Armonk, NY, USA). When the difference in variance was >4, skewness was <-2, or kurtosis was >2, the data were considered non-parametric and the Kolmogorov–Smirnov test was performed. Correlations were undertaken by performing Pearson's correlation tests in SPSS. Parallelism of the regression lines test was undertaken using SAS.

## 3. Results

### 3.1. TNA-Cifer Reagent E rapidly inactivates SARS-CoV-2

**Figure 1:**
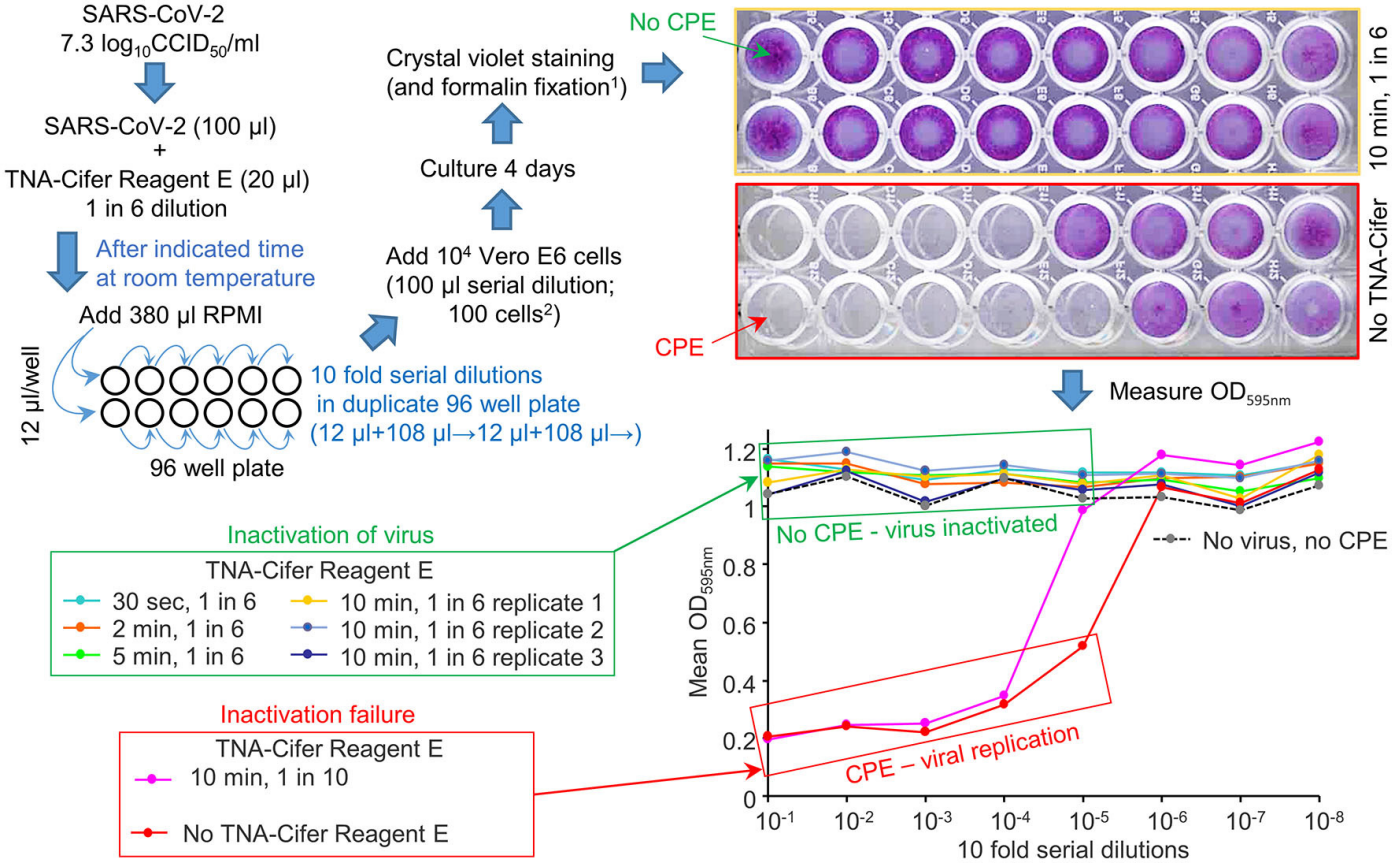
TNA-Cifer Reagent E inactivation of SARS-CoV-2. SARS-CoV-2 in RPMI 1640 supplemented with 2% FBS (RPMI) was treated with TNA-Cifer Reagent E and virus inactivation determined by CCID_50_ assays. The replication-competent virus killed the Vero E6 cells in the 96-well plates via cytopathic effects (CPEs). When the virus was inactivated, there was no CPE, and the Vero E6 cells were stained blue using crystal violet (top right, yellow border); the crystal violet dye then provided a high OD 595 nm (expressed as a mean of duplicates; bottom right, green box). In the absence of the virus, but during the presence of TNA-Cifer Reagent E, there was no CPE (bottom right, dashed black line). At high dilutions (e.g., bottom right, 10^−6^-10^−8^), the virus was diluted out, and there was no CPE; thus, the OD was high. ^1^Formalin fixation allows release from PC3/BSL3. ^2^The final dilution of TNA-Cifer Reagent E was 1 in 500 in the first two wells of the CCID_50_ assay titration.

To investigate the ability of TNA-Cifer Reagent E to inactivate SARS-CoV-2, a SARS-CoV-2 stock (7.3 log_10_CCID_50_/ml) was treated with TNA-Cifer Reagent E for 30 s or 2, 5, or 10 min at room temperature; then, the presence of replication-competent virus was determined by CCID_50_ assays in Vero E6 cells. The presence of replication-competent virus is revealed by virus-induced cytopathic effects (CPEs), resulting in the death of the Vero E6 cells and, thus, the loss of crystal violet staining. The reduced crystal violet staining is then measured by spectrophotometry, resulting in low OD_595nm_ readings. Conversely, inactivated virus does not induce CPEs, thereby providing high OD_595nm_ readings (Yan et al., 2021) (Figure 1).

No CPE was detected after treating the virus with a 1-in-6 dilution of TNA-Cifer Reagent E and incubating with the buffer for any time point >30 s (Figure 1, green boxes: 30 s, 2, 5, and 10 min). The treatment with a 1-in-10 dilution of TNA-Cifer Reagent E followed by a 10-min incubation was not sufficient to inactivate the virus (Figure 1; red boxes, pink line). In the absence of the virus, and mock treatment with 1-in-6 TNA-Cifer Reagent E, no CPE was observed (Figure 1; No virus, no CPE), illustrating that TNA-Cifer Reagent E was not toxic to cells (i.e., it did not show false-positive CPEs).

These data illustrate that adding 1 part TNA-Cifer Reagent E to 5 parts of the medium containing SARS-CoV-2 resulted in the inactivation of the virus after 30 s.

### 3.2. RT-qPCR after column-based purification vs. TNA-Cifer Reagent E extraction

**Figure 2:**
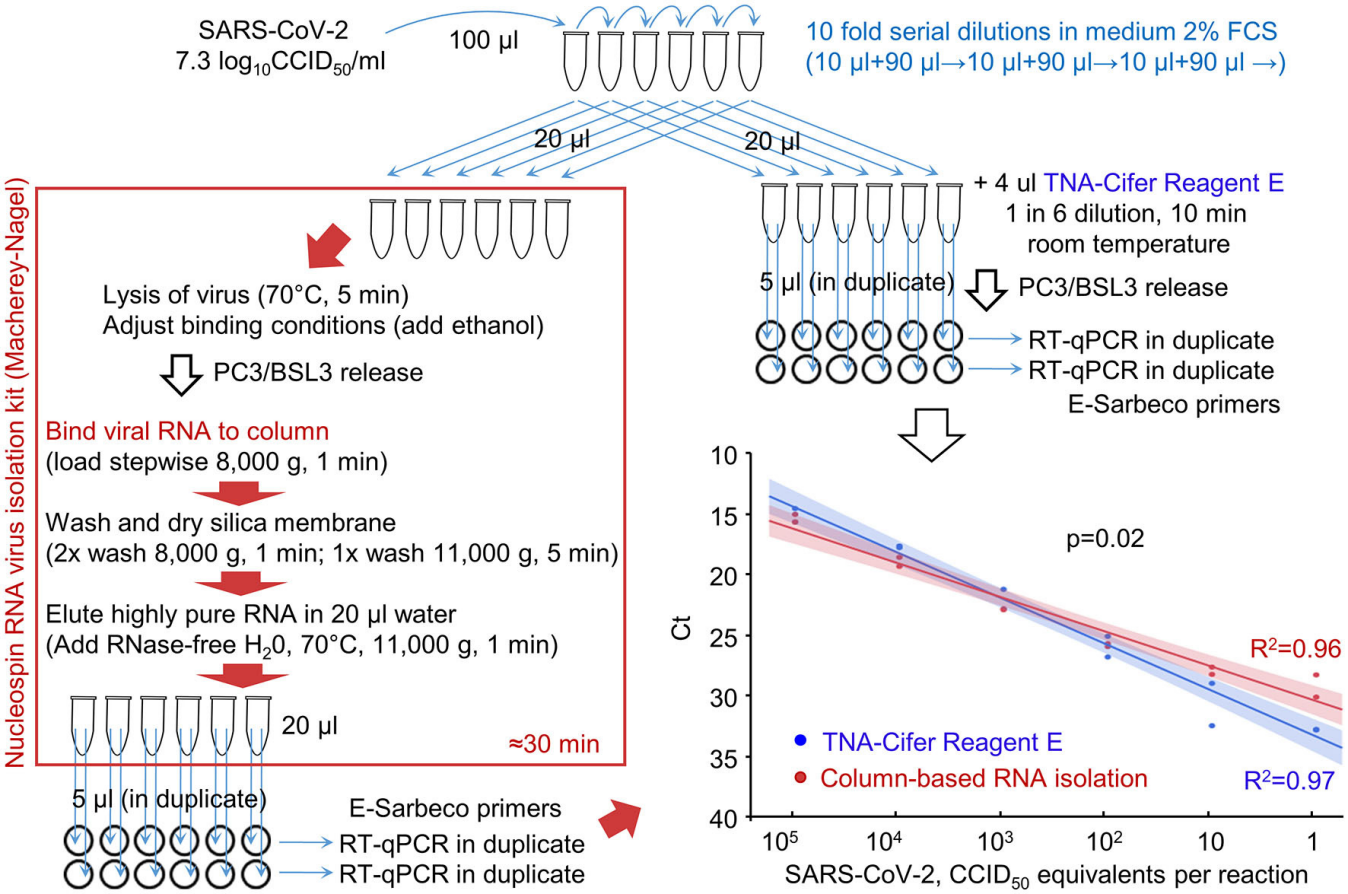
RT-qPCR results after RNA extraction from SARS-CoV-2 dilutions using TNA-Cifer Reagent E vs. a column-based RNA isolation kit. RNA was extracted from serial dilution of SARS-CoV-2 using either a column-based RNA-virus isolation kit (red/brown box) or TNA-Cifer Reagent E (top right). Graph bottom right; the samples were analyzed by RT-qPCR, and the Ct values were provided for each dilution. Each of the duplicate samples was tested in duplicate by RT-qPCR, with Ct values averaged to provide a single data point. The lines (bottom right) represent logarithmic regression line fits, with 95% confidence intervals. *R*^2^–coefficient of determination. The *p*-value obtained by performing the parallelism of regression lines test using the means of the duplicates is shown on the graph.

The ability of RT-qPCR to quantify viral RNA that was (i) purified using a standard column-based method or (ii) extracted using TNA-Cifer Reagent E was compared using serial dilutions of the virus (Figure 2). Plotting the cycle threshold (Ct) values against the viral dilutions provided regression lines with high coefficients of determination for both methods (Figure 2, *R*^2^). The slope for TNA-Cifer Reagent E was slightly, but significantly, steeper (*p* = 0.02), indicating that the ability to discriminate between high- and low-RNA levels was slightly better for viral RNA extracted using TNA-Cifer Reagent E.

This experiment illustrated that TNA-Cifer Reagent E treatment can replace standard column-based viral RNA purification and can prepare samples for RT-qPCR without significant loss of performance.

### 3.3. Diagnostic performance evaluation using human nasal swab samples

**Figure 3:**
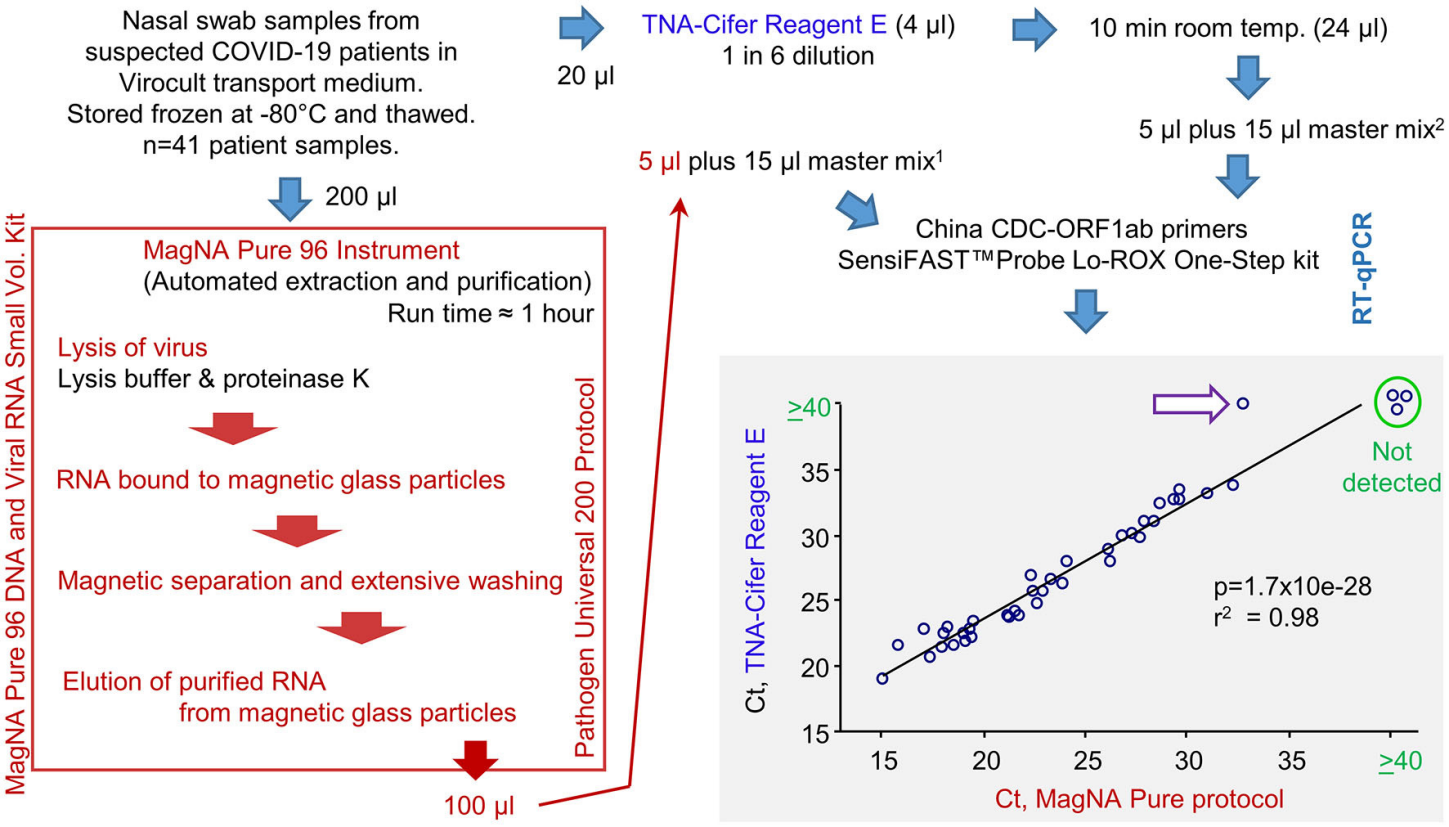
RT-qPCR results for human nasal swab samples extracted using MagNA Pure 96 protocol vs. TNA-Cifer Reagent E. Viral RNA was extracted from human nasal swabs following a standard diagnostic protocol using the MagNA Pure 96 instrument or by treatment with TNA-Cifer Reagent E. Viral RNA was then added to RT-qPCR using China CDC-ORF1ab primers. Ct values are shown and analyzed by Pearson correlation, with the coefficient of determination and significance shown. Ct values ≥40 were deemed negative (not detected). A single sample (purple arrow, white fill) was negative after TNA-Cifer Reagent E extraction but positive when the MagNA Pure 96 protocol was followed. ^1^5 μl represents 10 μl of the original Virocult transport medium. ^2^5 μl represents 4.17 μl of the original Virocult transport medium. This provides a conservative correction (≈240%) for potential RNA losses (up to ≈60%) during the MagNA Pure 96 procedure so that more input RNA for RT-qPCR would not be used for the TNA-Cifer Reagent E than for the MagNA Pure 96 samples.

A total of 41 frozen swab samples from suspected COVID-19 patients, collected in the Sigma-Virocult virus transport medium (VTM), were provided by Pathology Queensland. These samples were thawed and RNA-extracted using either (i) automated extraction using the MagNA Pure 96 instrument, with the Pathogen Universal 200 protocol setting, or (ii) TNA-Cifer Reagent E (Figure 3; Supplementary Table 1). RNA samples were added to RT-qPCR master mixes containing the China CDC ORF1ab primer sets (Niu et al., 2020), and the Ct values for the MagNA Pure extraction/purification are compared with TNA-Cifer Reagent E treatment (Figure 3, bottom right).

The correlation between the two methods was highly significant, with only one sample negative for TNA-Cifer Reagent E but positive for MagNA Pure (Figure 3, purple arrow white fill). This false negative had the highest Ct value (32.8) of all the MagNA Pure samples. To allow for RNA losses during the MagNA Pure protocol, an equivalent of 10 μl of the original Virocult medium was used in the RT-qPCR. This was compared with 4.1 μl of the medium used for TNA-Cifer Reagent E extracted samples (Figure 3, see legend). The discrepancy (false negative) may thus have arisen from overcompensation for the potential RNA losses (i.e., ≈240% for a 60% loss) for the MagNA Pure protocol and/or the low optimal performance of TNA-Cifer Reagent E in conjunction with the Virocult viral transport medium (see below). The latter is also apparent from the mean increase of 3.16 in Ct values for the TNA-Cifer Reagent E treated samples (Supplementary Table 1). A set of 20 negative samples from healthy controls were also tested, with none of these showing Ct values ≤40.

The sensitivity and specificity data for TNA-Cifer Reagent E prepared samples are shown in Table 1. The calculations are based on 37 true positives and 1 false negative, with 23 true negatives.

**Table 1:** Sensitivity and specificity for RT-qPCR diagnosis for human nasal swab samples treated with TNA-Cifer Reagent E (from Figure 3), calculated as described (see Medcalc-Website).

**Statistic**	**Value**	**95% CI**
Sensitivity	97.37%	86.19%−99.93%
Specificity	100%	85.18%−100%
Negative likelihood ratio	0.03	0.00–0.18
Positive predictive value	100%	
Negative predictive value	95.83%	76.88%−99.38%
Accuracy	98.36%	91.20%−99.96%

Sample size n = 61; 41 patients samples and 20 negative controls (Supplementary Table 1).

### 3.4. Storage of SARS-CoV-2 samples in TNA-Cifer Reagent E

**Figure 4:**
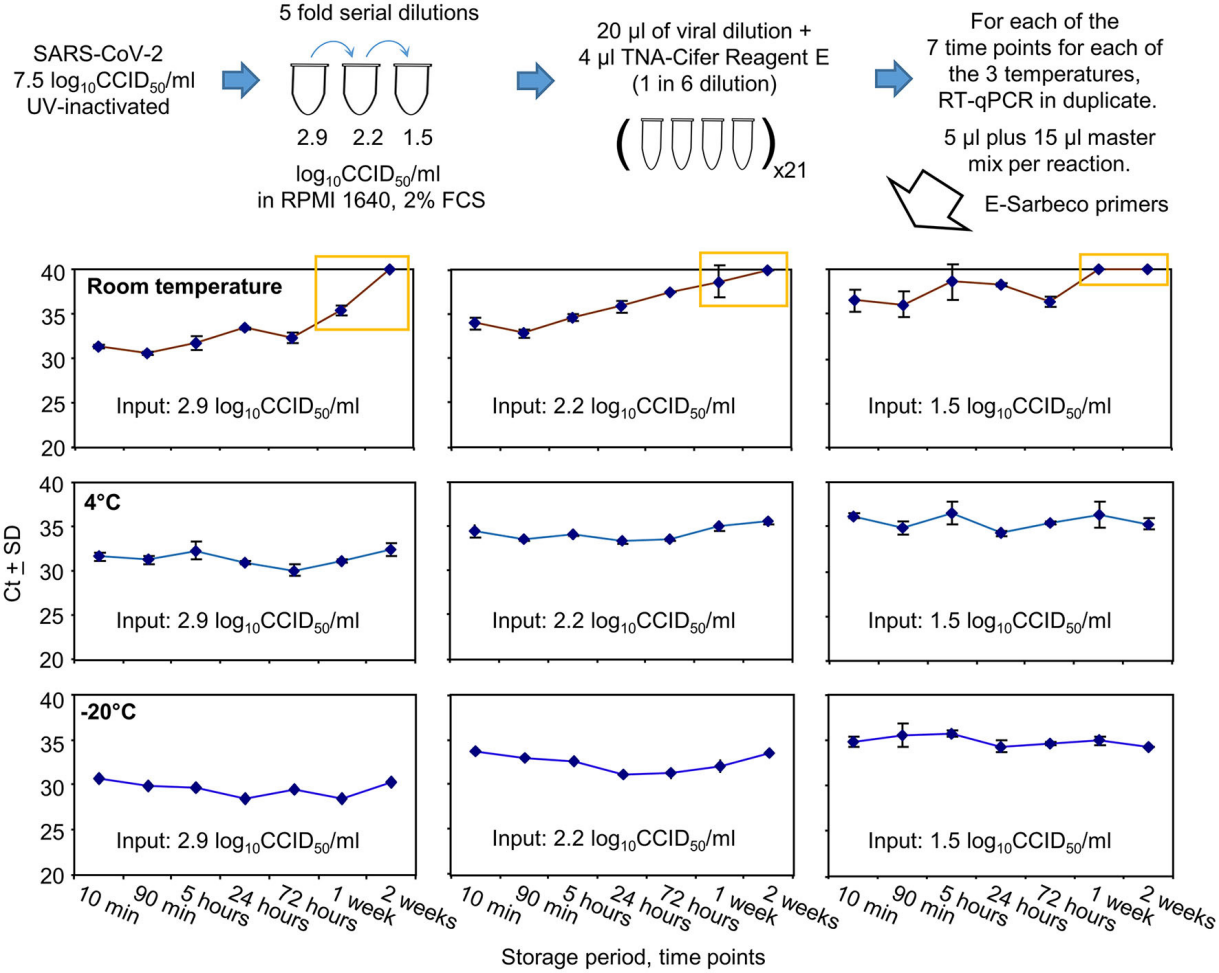
SARS-CoV-2 RNA detection after TNA-Cifer Reagent E treatment and storage at different times and temperatures. UV-inactivated SARS-CoV-2 virus was diluted in RPMI supplemented with 2% fetal bovine serum (FBS) and treated with TNA-Cifer Reagent E. It was kept at different temperatures for the indicated periods of time and was then analyzed by RT-qPCR. Yellow boxes indicate where Ct values had reached ≥40 (deemed to be not detected) or increased by >4 relative to the Ct values for the 10-min time point. All RT-qPCR results for the 0.8 log_10_CCID_50_/ml had Ct values ≥40. All the −20°C samples underwent one freeze-thaw cycle prior to RT-qPCR.

UV-inactivated SARS-CoV-2 was serially diluted in RPMI 1640 supplemented with 2% fetal bovine serum (FCS) and treated with TNA-Cifer Reagent E (1 in 6), with the mixtures stored at three different temperatures for seven different time periods (Figure 4). The mixtures were then analyzed in duplicate by RT-qPCR using the E-Sarbeco primer set. All RT-qPCR results for the 0.8 log_10_CCID_50_/ml dilution had Ct values ≥40 (not shown); thus, 1.5 log_10_CCID_50_/ml represented the lowest level of reliable detection for this series of five-fold serial dilutions (Figure 4).

Storage at room temperature for a week or longer resulted in Ct values increasing by 4 or reaching ≥40 (Figure 4, yellow boxes). For the lowest dilution (1.5 log_10_CCID_50_/ml), the Ct results also showed a loss of a consistent trend and increased variance at and beyond 5 h (Figure 4). Storage at 4°C or −20°C showed no increase in Ct values ≥1.07 at any time point (Figure 4, 4°C and −20°C).

In summary, storage in TNA-Cifer Reagent E (1 in 6) at 4°C or −20°C for up to 2 weeks did not significantly affect the detection of RT-qPCR. For samples stored at room temperature, a substantial loss of viral RNA occurred after 1 week. For samples with low levels of viral RNA, RT-qPCR remained detectable for 3 days, but the results varied after 90 min.

### 3.5. Performance of TNA-Cifer Reagent E with different transport media

**Figure 5:**
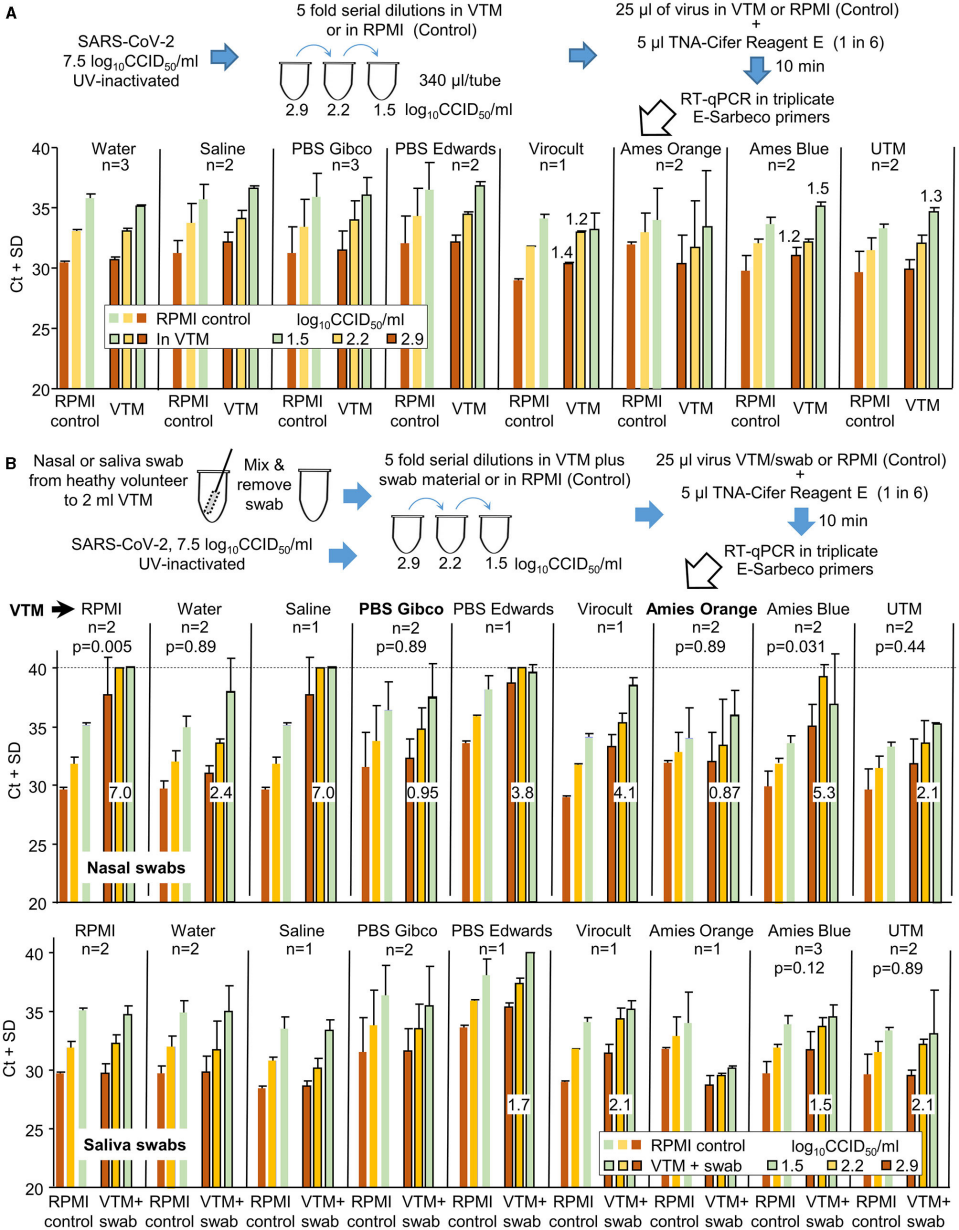
The compatibility of different viral transport media (VTM) with TNA-Cifer Reagent E. **(A)** SARS-CoV-2 was UV-inactivated, permitting release from PC3/BSL3 and allowing the experiment to be conducted under PC2/BCL2 biocontainment. The UV-inactivated virus was diluted in various VTM, mixed with TNA-Cifer Reagent E, and then evaluated by RT-qPCR. For *n* = 2 or 3, the mean and SD of two or three independent experiments are shown, with RT-qPCR undertaken in triplicate for each experiment. For *n* = 1, the mean and SD of the triplicates of one experiment are shown. The increase in Ct is provided above the bar for increases in Ct >1 relative to the same amount of virus in the side-by-side RPMI controls (in the same RT-qPCR run). RPMI control represents the RPMI 1640 medium supplemented with 2% FCS. **(B)** As for A, except for nasal or saliva swabs (from healthy volunteers), all others were added to the VTM prior to their use in virus dilutions. Swabs were not added to the side-by-side RPMI controls (n as for A). Numbers in white boxes represent the mean increase in Ct values when compared to the RPMI controls, expressed as “the mean Ct across the three viral dilutions for the VTM” minus “the mean Ct across the three viral dilutions for RPMI.” For nasal swabs (top), only PBS Gibco and Amies Orange (in bold) had increases in Ct values below 1. As many of the datasets were non-parametric, the Kolmogorov–Smirnov test was conducted throughout to allow comparisons; for *n* = 2 datasets, 6 values for RPMI controls were compared with 6 values for VTM to provide the indicated *p*-values. For *n* = 3 experiments, this value was 9.

Both nasal and saliva swabs used to collect patient samples are generally transported to pathology laboratories in the viral transport medium (VTM). To investigate the compatibility of different VTM with TNA-Cifer Reagent E, UV-inactivated SARS-CoV-2 was diluted in different VTM and was tested side by side (in the same RT-qPCR run) with UV-inactivated SARS-CoV-2 diluted in RPMI 1640 supplemented with 2% FCS (Control). Increases in Ct values for all the VTM tested were ≤1.5 (Figure 5A). A change in Ct of 1.65 represents a 0.5 log decrease in detection sensitivity (Tom and Mina, 2020).

The same experiment was repeated, except that the UV-inactivated SARS-CoV-2 was diluted using VTM into which the material from nasal swabs (from healthy volunteers) had been added (Figure 5B). Only two VTM showed Ct increases ≤1.5, with changes also not reaching significance (Figure 5B, Nasal swabs, PBS Gibco, Amies Orange). When material from saliva swabs was added, four VTM showed small increases ≥1.5, with none showing significant differences (Figure 5B, Saliva swabs).

In summary, TNA-Cifer Reagent E is compatible with most VTM for saliva swabs, but for nasal swabs, each VTM requires individual evaluation for potential loss of sensitivity.

### 3.6. Performance of TNA-Cifer Reagent E with the Mic real-time PCR cycler

**Figure 6:**
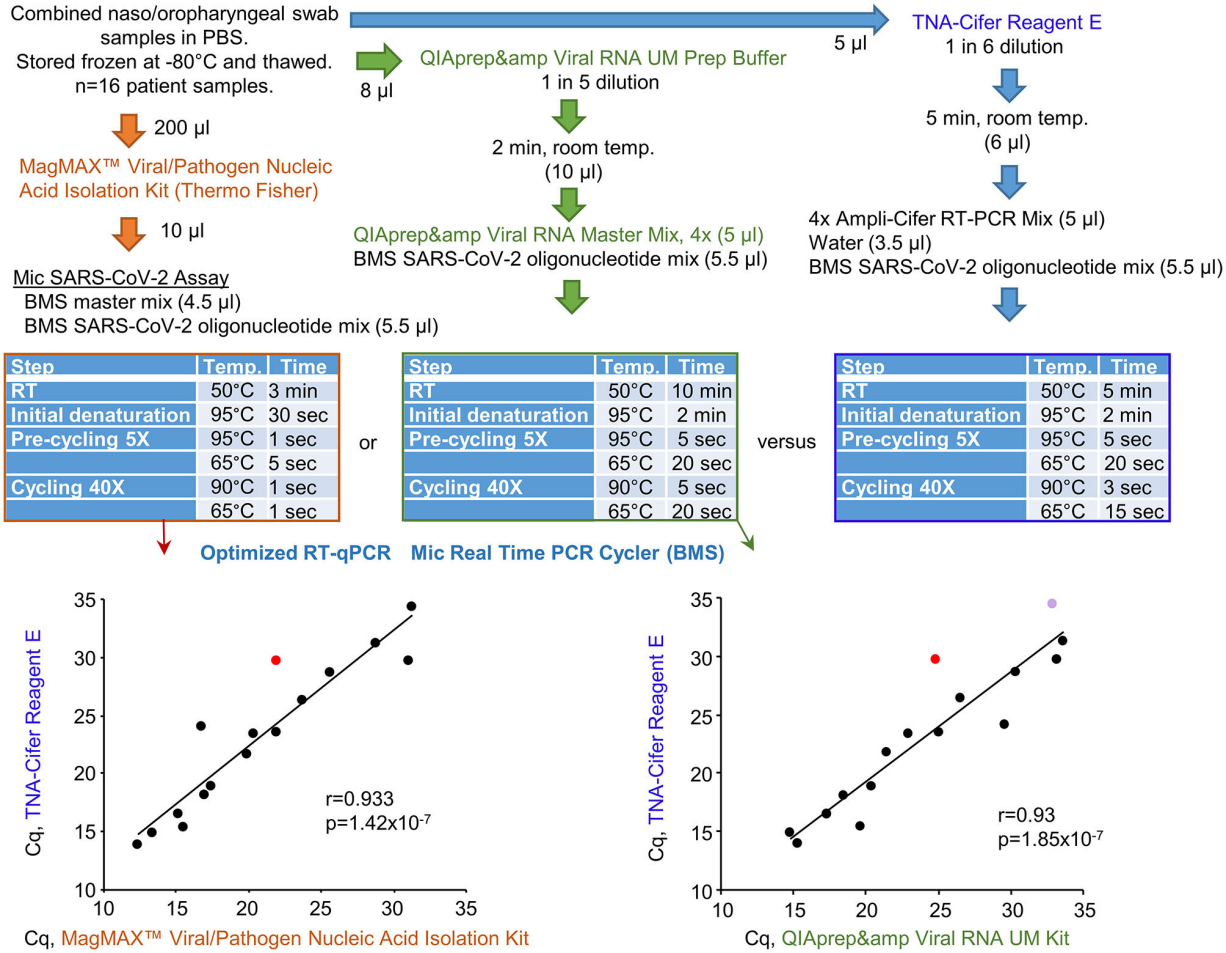
Performance of TNA-Cifer Reagent E with the Mic real-time PCR Cycler. RT-qPCR results for TNA-Cifer Reagent E treated combined naso/oropharyngeal swab samples from patients who tested positive for SARS-CoV-2 were compared with RT-qPCR using the MagMAX™ Viral/Pathogen Nucleic Acid Isolation Kit and the QIAprep&amp Viral RNA UM Kit. Each of the three methods used the proprietary oligonucleotide mix and the Mic real-time PCR Cycler from Bio Molecular Systems (BMS). The cycling conditions (blue tables) and final reagent concentrations were optimized for each method for use with the proprietary oligonucleotide mix and the Mic real-time PCR Cycler. Efficiency percentages were >70%, except for one RT-qPCR result for a sample treated with TNA-Cifer Reagent E (red) and another RT-qPCR result for a sample tested using the QIAprep&amp Viral RNA UM Kit (purple). Statistics by Pearson correlations providing the correlation coefficients and *p*-values.

Bio Molecular Systems (BMS) has developed a relatively inexpensive and portable Mic real-time PCR Cycler and Myra automatic robotic handler (see Bio-Molecular-Systems-Website, 2022). COVID-19 patient swab samples stored in PBS were tested using three protocols that all utilized a BMS proprietary oligonucleotide mix, the Myra automatic robotic handler, and Mic real-time PCR Cycler (Figure 6). RT-qPCR results for TNA-Cifer Reagent E treated samples were compared with (i) RNA-extracted and purified samples using the MagMAX™ Viral/Pathogen Nucleic Acid Isolation Kit and (ii) the QIAprep&amp Viral RNA UM Kit (Fenaux et al., 2022). The RT-qPCR conditions for each of the three systems were independently modified for optimal performance with the BMS oligonucleotide mix and Mic real-time PCR Cycler (Figure 6, blue tables). The Mic real-time PCR Cycler provides an efficiency percentage (Ruijter et al., 2009), with values >70% deemed to represent a valid test. High levels of correlation for Cq results were obtained across the 16 samples, although one sample treated with TNA-Cifer Reagent E and another sample tested using QIAprep&amp Viral RNA UM Kit RT-qPCR test gave efficiency percentages <70% (Figure 6, graphs). These results illustrate that, after optimizing the cycling conditions for a new system, the performance of TNA-Cifer Reagent E sample preparation for RT-qPCR is very similar to established methodologies.

## 4. Discussion

In this study, we demonstrated the utility of the TNA-Cifer Reagent E in RT-qPCR testing of SARS-CoV-2. This buffer has the ability to rapidly inactivate the virus while also enabling the sample to be directly added to RT-qPCR reactions. A sensitivity of 97% and specificity of 100% (Table 1) compares very favorably with established RNA extraction and purification processes, although the optimal performance for nasal swab samples was only obtained for two VTM (PBS Gibco and Amies Orange). The ability to store TNA-Cifer Reagent E treated samples for up to 2 weeks at 4°C means that retesting can be undertaken on the same samples without the repeat test being compromised by RNA degradation issues. Finally, we illustrated that, after optimizing the cycling conditions, TNA-Cifer Reagent E performed as well as established methods when using the Myra automatic robotic handler and Mic real-time PCR Cycler system (BMS).

The ability of the TNA-Cifer Reagent E to inactivate SARS-CoV-2 and provide a “direct to RT-qPCR” process also has utility in medical research settings. For instance, all infectious materials must ordinarily be inactivated by a validated process before they can be released from a PC3/BSL3 biocontainment facility into a standard laboratory setting (often PC2/BSL2). The treatment of infected samples with TRIzol Reagent (containing phenol and guanidine isothiocyanate) is currently a widely used inactivation and release methodology, but it requires a series of steps (phase separation, precipitation, washing, and resuspension), which can be time-consuming, especially when there are a large number of samples (Rawle et al., 2021; Van Oosten et al., 2021; Guimond et al., 2022).

A limitation of this study is that not all SARS-CoV-2 variants of concern were evaluated. However, the use of a chemical inactivation and extraction process, such as TNA-Cifer Reagent E, is not likely to be significantly influenced by amino acid changes in the viral proteins. Although the inactivation of the Hendra virus, Nipah virus, and dengue virus has been demonstrated (Pollak et al., 2022b, 2023a,b), the inactivation of common human pathogens, such as HIV and hepatitis viruses, has not yet been demonstrated, with such viruses being an established safety concern for blood tests. The influence of VTM and swabs on the ability of TNA-Cifer Reagent E to inactivate SARS-CoV-2 was also not evaluated, with SARS-CoV-2 inactivation potentially influenced by different excipients and their interactions with various patient-derived materials. The reasons underpinning the increases in Ct values with certain VTM (e.g., Supplementary Table 1) also remain to be established. The ability of TNA-Cifer Reagent E-treated swab samples to allow multiplex RT-qPCR testing for a panel of respiratory viruses (e.g., influenza, respiratory syncytial virus, and human metapneumovirus) remains to be evaluated. The next step is clinical field evaluations of TNA-Cifer Reagent E in conjunction with specific emerging RT-qPCR-based technologies, such as the relatively inexpensive portable Myra and Mic instruments (BMS). Such evaluations should be performed in settings where viral inactivation is important (e.g., limited access to BSL2 facilities), immediate results are paramount (e.g., for the timely intervention of vulnerable patients with Paxlovid treatment therapy), and access to automated RNA extraction equipment is limited or associated with excessive costs or time delays.

## Data availability statement

The raw data supporting the conclusions of this article will be made available by the authors, without undue reservation.

## Ethics statement

The studies involving humans were approved by the HREC of the Royal Children's Hospital, Brisbane, Australia approved use of deidentified human nasal and saliva swabs (Enhanced Characterisation of Respiratory Virus Infections LNR/19/QCHQ/49476). The studies were conducted in accordance with the local legislation and institutional requirements. The human samples used in this study were acquired from A bank of 41 frozen swab samples from suspected COVID-19 patients, collected in Sigma-Virocult virus transport medium (VTM), were provided by Pathology Queensland. Written informed consent for participation was not required from the participants or the participants' legal guardians/next of kin in accordance with the national legislation and institutional requirements.

## Author contributions

NP: methodology, validation, formal analysis, resources, data curation, and writing—review and editing. DR: investigation, validation, resources, supervision, and methodology. KY, TL, and RL: investigation and methodology. CB, CW, and KH: investigation. NE: investigation and review. NC and MH: methodology, validation, and data curation. DW: resources, supervision, and data curation. AS: resources, methodology, visualization, and writing—review and editing. JM: conceptualization, investigation, methodology, validation, formal analysis, resources, data curation, supervision, and writing—review and editing. All authors contributed to the article and approved the submitted version.
